# The effect of cigarette smoking on first‐trimester crown–rump length

**DOI:** 10.1002/ajum.12313

**Published:** 2022-08-23

**Authors:** Nicole Stamatopoulos, Chuan Lu, Samuel Vo, Mercedes Espada Vaquero, Mathew Leonardi, George Condous

**Affiliations:** ^1^ Acute Gynaecology, Early Pregnancy and Advanced Endoscopy Surgery Unit, Sydney Medical School Nepean University of Sydney, Nepean Hospital Penrith New South Wales Australia; ^2^ Women and Children's Health Services Nepean Hospital Kingswood New South Wales Australia; ^3^ Department of Computer Sciences Aberystwyth University Wales UK; ^4^ Department of Obstetrics and Gynecology McMaster University Hamilton Ontario Canada

**Keywords:** cigarette smoking, fetal growth, first‐trimester pregnancy, growth restriction

## Abstract

**Objectives:**

Does cigarette smoking impact the embryonic growth rate in the first trimester?

**Methods:**

This is a retrospective multicentre observational study of 2912 pregnancies. Women who presented to the early pregnancy and perinatal ultrasound units between 2010 and 2019 were included in the study. The data collected included the following: smoking status, the crown–rump length (CRL) of the pregnancy at the first ultrasound that showed an embryonic heart rate, the gestation in days and the CRL at another ultrasound up to the nuchal translucency scan and the gestation in days. Additional demographic data included the following: age, weight, height, parity and mode of delivery.

Of the 2912, complete smoking and demographic data were available for 657 pregnancies. One hundred and thirty‐seven (26.3%) were smokers, and 520 (73.7%) were not. The rate of change of smokers vs non‐smokers between two CRLs and two different days of gestation was calculated. The Wilcoxon rank sum test with continuity correction was used for statistical analysis.

**Results:**

This gives a value of *W* = 31,940 and a P‐value = 0.06. There is a slight shift in location for the smokers; however, it is not statistically significant. The insignificance may be due to the general large variance in growth rate.

**Conclusion:**

The impact of cigarette smoking on embryonic growth rate detected by CRL in the first trimester is statistically insignificant.

## Introduction

Cigarette smoking is a worldwide major public health issue and a cause of substantial morbidity and mortality.^1^ It is the most common single preventable reason for infant morbidity and mortality and is associated with low infant birthweight compared with non‐smokers.^2^, ^3^ Smokeing during pregnancy has been linked to socio‐economic disadvantage and engaging in risky behaviour that may be linked to low birthweight.^4^

The critical smoking exposure window and timing of smoking‐related fetal growth restriction are limited. There is uncertainty about the impact of smoking cessation in early pregnancy, and the extent to which fetal growth restriction can be prevented or minimised by lowering cigarette consumption. Few studies having explored when during pregnancy smoking starts to effect growth.^4^ The earliest gestation that has investigated the impact of cigarette smoking on the crown–rump length (CRL) in the first trimester is between the 10^th^ and 13^th^ week of gestation.^5^

It is known that cigarette smoking has a negative effect on reproductive organ health and decreases oestrogen secretion.^1^

Pregnancy is a critical period at which successful smoking cessation interventions may not only enhance the health of mothers and babies but also disrupt the familial propagation of smoking.^2^

This study aimed to assess whether the impact of cigarette smoking on embryonic growth rate can be detected by CRL measurement in the first 12 weeks.

## Methods

**Figure 1:**
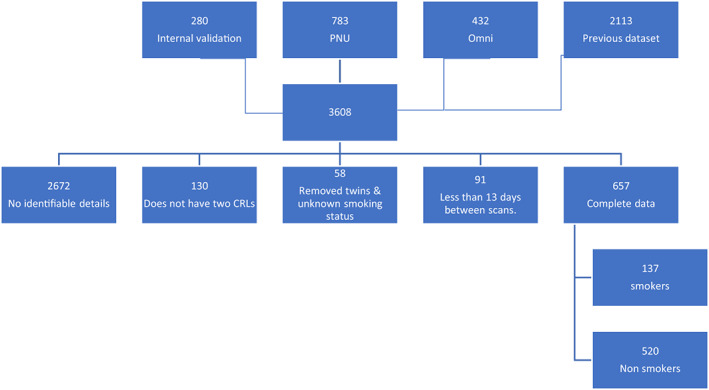
Flow diagram for patient inclusion criteria.

This is a retrospective multicentre observational study of 3608 pregnancies. Data from three units were reviewed. The units were as follows: the early pregnancy unit (with a current and previously maintained dataset) and perinatal ultrasound unit from Nepean Hospital in western Sydney and a private practice, Omni in Northern Sydney. The date range was from November 2001 to July 2019. The viewpoint reporting system was used to search for the early pregnancy ultrasound reports. The keywords used for the search in viewpoint were ‘early pregnancy’, ‘viable’ and ‘dating’. A total of 1495 new pregnancies were identified, and the reports were reviewed in addition to the previous 2113 from the previous dataset (Figure 1).

The data that were collected included the smoking status of the women and demographic data including the following: height, weight, parity, mode of delivery and age (Table 1).

**Table 1:** Demographic data comparing smokers and non‐smokers

n	Smoker (137 cases)	Non‐smoker (520 cases)
Average age (years)	26.14	28.76
Average height (cm)	163.56	164.11
Average weight (kg)	75.83	74.14
Average BMI (kg/cm^2^)	28.35	27.53
Average parity	1.1	0.88
Nulliparous	39.4% (54/137)	42.7% (221/517)
Para 1	28.5% (39/137)	36.9% (191/517)
Para 2+	32.1% (44/137)	20.3% (105/517)
Spontaneous pregnancies based on available data	97.0% (129/133)	93.4% (470/503)

Sonographic data included the following: CRL, gestational sac in three orthogonol planes and the gestational age in days according to the ultrasound at two different time points in the first trimester. The first time point was usually at 6–8 weeks, and the second was the nuchal translucency scan at 11 and a half to 13 weeks. This data and the previously mentioned data were collated in a Microsoft Excel spreadsheet. The ultrasound CRL and gestation in days at both time points were analysed.

Low negligible risk ethics approval was granted through Nepean Blue Mountains Local Health District Human Research Ethics Committee (EC00151) on 1^st^ November 2016. The Wilcoxon rank sum test with continuity correction was performed.

## Statistical analysis

The growth rate (mm/day) was calculated using the difference between CRL at the nuchal translucency ultrasound and CRL at the dating ultrasound scan (in mm) and dividing this difference by the difference in days between both ultrasound scans (days).

The mean rate of growth in the smoker group was 1.40 mm/day (SD 0.17) compared with the mean rate of growth in the non‐smoker group of 1.44 m/day (SD 0.22).

The distribution of both the smoking and the non‐smoking groups for the growth rates was reviewed and followed an asymmetric distribution. The Wilcoxon rank sum test was performed which is nonparametric, and the assumption of normality was not required. It also tests the ordering of data and analysing with many outliers. The Wilcoxon rank sum test was then performed with continuity correction, and an alternate hypothesis that the true location shift is not equal to 0. It compared the two independent points of CRL growth.

## Results

**Figure 2:**
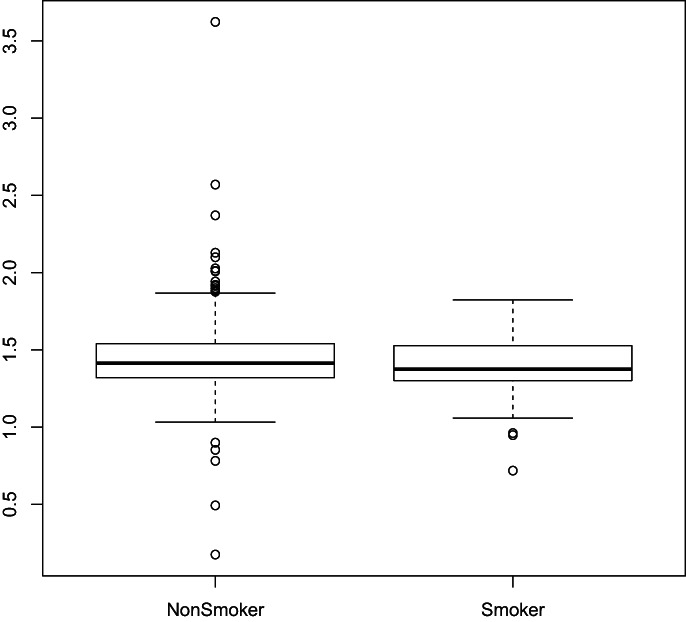
Box plot of non‐smoker vs. smoker.

**Figure 3:**
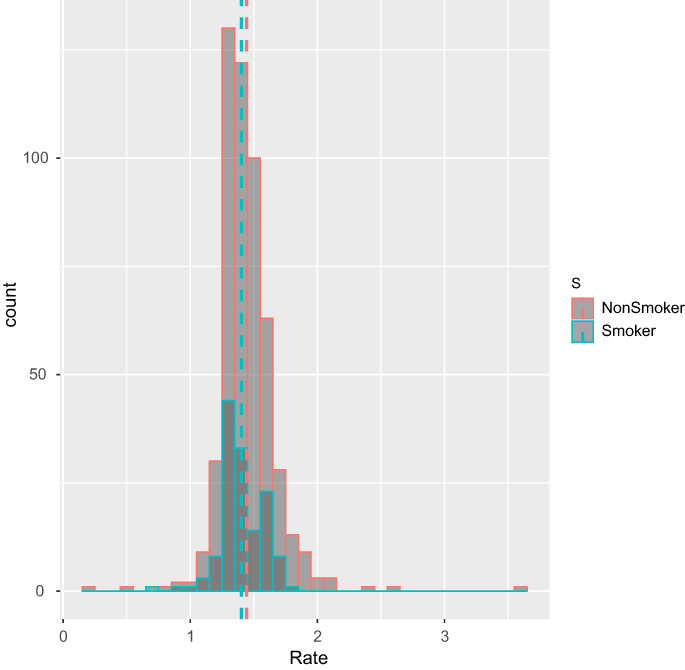
Column chart of smoker vs. non‐smoker.

**Figure 4:**
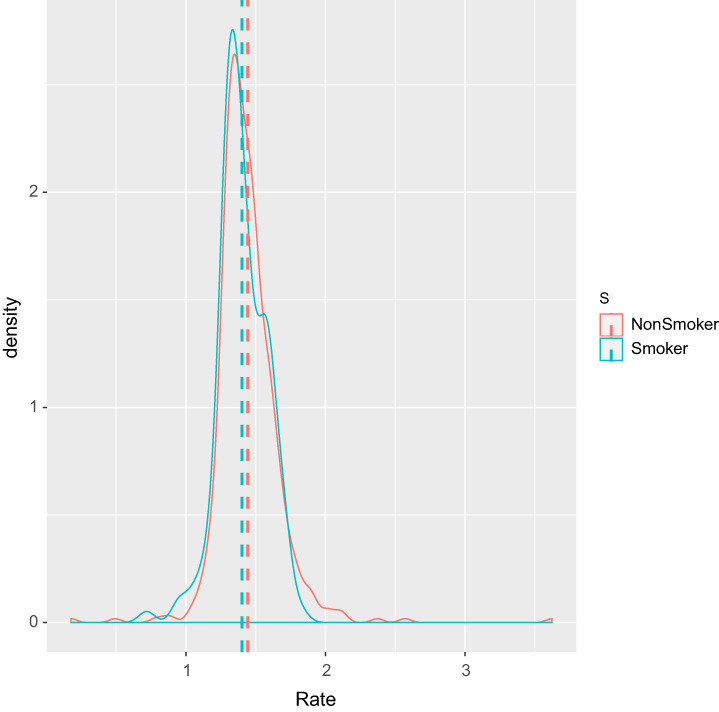
Line graph of smoker vs. non‐smoker.

The data rate by smoker was *W* = 31,940 and a P‐value of 0.062. Figures 2, 3, 4 show these results in a box plot, column and line chart, respectively. While there appears to be a visual difference in the growth rate between smokers and non‐smokers, with a slower growth rate in smokers, the difference is not statistically significant.

## Discussion

This study does not show a statistically significant impact of cigarette smoking on embryonic growth rate by CRL in the first 13 weeks of pregnancy. There are very few studies assessing fetal growth despite research showing that maternal smoking limits fetal growth.^6^ Nicotine interacts with receptors in placental vasculature resulting in decreased placental blood flow and fetal vasoconstriction, which leads to a disruption of the delivery of oxygen and nutrients to the fetus. This reduced blood flow leads to fetal malnutrition and is thought to be a causal mechanism for the effects of prenatal smoking exposure on poor fetal growth.^7^ This shows the association between the limitation in growth being linked to placental function.^3^ This has led to a dose dependent proportionally higher number of abnormal placentas.^6^

First‐trimester measurements such as the gestational sac (GS), yolk sac (YS) and CRL are often performed to assess for pregnancy loss or viability.^8^, ^9^ The first ultrasound measurement after the CRL to measure the fetus is the biparietal diameter (BPD). It is measured from 12 to 14 weeks as part of first‐trimester screening^10^ or an early structural scan with cell‐free DNA‐based screening.^11^

Interestingly, a Saudi Arabian study showed a relationship with cigarette smoking and low placenta associated plasma protein (PAPP) A.^12^ PAPP A is expressed at high levels in the first trimester. If they are low, there is an association with low birthweight.^13^ Thereafter, fetal growth assessment is measured with head circumference, BPD, abdominal circumference and femur length.^14^

Fetal growth beyond the first trimester is assessed using both ultrasound measurements: Doppler flow velocity waveforms^15^ and fetal biometry.^6^ These measurements are made after the first trimester with the measurement of the umbilical and uterine artery Dopplers^6^ and BPD.^10^, ^11^

Cigarette smoking may statistically begin to affect fetal growth when uterine artery Doppler flow studies can first be assessed at 11 weeks' gestation. At this gestation, this measurement has been used to predict which pregnancies may be affected by pre‐eclampsia and/or fetal growth restriction (FGR). However, growth restriction did not, in this context, consider cigarette smoking as the cause of FGR.^16^

An assessment of placental function was conducted in a study in 2009 in Australia and New Zealand. It compared umbilical and uterine artery Doppler waveforms and fetal size in smokers vs non‐smokers. Smokers had a statistically significant higher umbilical resistance index at 20 weeks, even when adjusted for confounders. This measurement was used as a surrogate measure for an abnormal placental villous vascular tree. They concluded that this may contribute to later fetal growth restriction in women who smoke.^17^ Little is known, however, regarding the underlying mechanism in restricting fetal growth.^6^

Other research indicates that there is a decrease in longitudinal and intra‐abdominal organ growth of a fetus with mothers who smoke.^6^ Smoking also appears to preferentially affect peripheral muscle mass. These changes appear to be prior to 33 weeks.^18^

Maternal smoking increases the risk of FGR and postnatal effects including ischaemic heart disease, hypertension and diabetes.^19^

Limitations in this study include the self‐reported smoking amounts. Without a more accurate measurement of smoking, the dose response will never be able to be assessed. More intensive measures and biological assays may better assess maternal use and fetal exposure.^7^

Other studies suggest micronutrient deficiencies exist in women who smoke in pregnancy. However, it is unknown whether this is due to increased requirements, lower dietary supplement intake or other factors.^20^

CRL may not be the correct tool to detect minor fetal growth changes related to smoking. Therefore, a small effect may not be easily observable.

There is also the limitation with the attrition rate in this study. While the initial number of patients is close to 3000, the dataset is incomplete for many of the patients.

There is no statistical impact of cigarette smoking on embryonic growth rate that can be detected by CRL measurement in the first 13 weeks of pregnancy. Encouraging women to quit smoking in the first trimester is still beneficial for fetal growth.

## Ethics Statement

This material is my own original work, which has not been previously published elsewhere. The paper is not currently being considered for publication elsewhere. The paper reflects my own research and analysis in a truthful and complete manner.

## Authorship statement

The material is the authors' own original work and has not been previously published elswhere.

## Funding

No funding information is provided.

## Conflict of Interest

There are no conflicts of interest.
